# Supplementary material for: Are carbon emissions trading and green financial instruments synergistic? -Comprehensive quantitative research based on content analysis
Source: PLoS One. 2024 Mar 7;19(3):e0298601. doi: 10.1371/journal.pone.0298601 (PMC10919601; doi:10.1371/journal.pone.0298601)
Supplement: S1 Data — (ZIP) [file pone.0298601.s001.zip › Data/地级市Z_F工作报告.zip!/╣π╢1⁄2/╖≡╔╜╩╨/└┤╫╘╣1⁄2╓┌║┼í╛┬φ┐╦╩2╛▌═°í┐_2004.docx]

各位代表：

我代表佛山市人民政府向大会作政府工作报告，请予审议，并请政协各位委员和其他列席人员提出意见。

2003年工作回顾

2003年是贯彻落实党的十六大精神的第一年，是我市新的行政管理体制全面运行的第一年，也是政府班子换届后的开局之年。一年来，市政府坚持以邓小平理论和“三个代表”重要思想为指导，深入贯彻落实党的十六大、十六届三中全会和胡锦涛总书记视察广东时的重要讲话精神，紧紧抓住发展这个第一要务，围绕市委第九次党代会提出的建设产业强市、文化名城和现代化大城市的目标，坚持一手抓抗击非典，一手抓经济建设，积极推进经济和社会各项事业全面发展，市第十二届人大一次会议确定的目标基本实现，为建设宽裕小康社会和率先基本实现社会主义现代化打下良好基础。

——经济持续健康协调发展。2003年预计全市生产总值1381.39亿元，比上年增长16.1%(绝对值为当年价，增长率按可比价计，下同)；人均生产总值40437元(按户籍人口计算)，增长14.7%。经济运行质量和效益进一步提高。全市税收总额273.11亿元，增长9.68%。其中：国税164.49亿元，增长5.74%；地税100.52亿元，增长16.2%。实现地方财政一般预算收入95.44亿元，增长15.87%。

——抗击非典取得阶段性胜利。面对非典疫情的袭击，把保护人民群众身体健康和生命安全放在第一位，沉着应对，科学决策，全力以赴，齐心协力，牢牢掌握防治非典工作主动权，深入开展防治非典知识普及、爱国卫生和全民健身运动，实现了非典病例高治愈率、低病死率。建立公共卫生、公共安全突发事件预警和应急处理机制，加强了卫生监督和疾病预防控制体系，确保全市人民身体健康。

——以规划引领经济社会向更高层次发展。先后编制完成并向社会公布产业发展、基础设施建设、教育发展、可持续发展的生态环境和建设文化名城等规划纲要；确定中心组团新城区、体育场馆等一批重点建设项目设计方案；组织实施交通干线公路网、电力建设、教育发展、可持续发展的生态环境和产业发展等项规划；各区在全市规划总体框架下，对规划进行了完善和修订。

——产业结构调整成效显现。第一产业完成增加值75.86亿元，增长3.8%；第二产业771.85亿元，增长20.5%；第三产业533.68亿元，增长12%。一、二、三产业的比重为5.555.938.6。农业和农村经济结构调整取得新进展。全市农业总产值148.82亿元，增长3.9%；农村经济总收入3448.8亿元，增长19.6%。农业生产条件改善，投入水利建设资金7.28亿元，完成水利工程219宗。工业产业整合聚集提升。全市完成工业总产值3297.25亿元，增长23.2%。十大优势行业快速发展，产值约占全市工业总产值的80%。名牌带动战略收到成效。民营经济实力壮大。规模以上民营企业工业产值1251.20亿元，增长33.9%，占全市规模以上工业总产值的48.5%；私营企业出口总值5.67亿美元，增长2.48倍；民营经济占国内生产总值比重达48%，成为推动全市经济增长的重要力量。现代服务业发展迅速。金融、商贸流通、房地产、电子商务等行业和连锁经营保持较快发展，专业市场和物流园区建设势头良好，会展经济取得较好效益。有效需求逐步扩大，全市社会消费品零售总额473.19亿元，增长12.7%。

——园区建设取得新进展。掀起“学江浙、促发展”和新一轮园区建设热潮，推行“一脑一体两翼”的园镇互动管理新体制，从市直机关选派一批优秀干部到重点工业园区任职，充实基层力量。工业园区整合力度加大，十大重点工业园区发挥主导作用，呈现出产业特色化。园区经济簇群效应显现，工业园区完成工业产值1250亿元，占工业总产值的37.9%。

——招商引资实现新突破。出台《关于进一步优化佛山投资环境，加大招商引资力度的若干意见》等政策措施，进一步优化投资环境。以园区为载体进一步创新招商引资方式，以引进世界500强等跨国公司和国内大企业为主攻方向，提高招商引资的质量和水平。成功举办9·26投资推介会，签约项目598项，投资总额641.6亿元。全市合同利用外资12.86亿美元，增长60.7%；实际利用外资12.4亿美元，增长23.7%，出口在实施多元化战略中实现历史性突破，出口总值102.23亿美元，增长29.6%。

——城市化进程步伐加快。按照建设两个一百万人口和五个三十至五十万人口的新城区构想，初步编制《城市发展概念规划》、《中心组团新城区规划方案》和《中心组团近期建设规划》。积极推进“并镇、并村、并校、减人”的改革，全市镇撤并率26.3%。十大建设工程项目正式启动。交通干线22项工程建设已经启动，城际快速轨道交通建设进展顺利，电源和电网建设加速推进。全社会固定资产投资总额422.51亿元，增长45.5%。城市资源有效整合。撤并28个路桥收费站，整合城市公交资源和邮政资源，市话号码成功升8位，统一全市电话区号，降低区间收费，满足电信发展需要，优化了投资环境，提升了城市形象。实行小额消费“一卡通”，利用信息网络和结算系统比较完善的优势，在五区推广电子钱包“佛山通”。

——文化名城建设全面启动。颁布建设文化名城规划纲要，文化事业和文化产业加快发展。“魅力佛山”文化系列活动蓬勃开展，“琼花焕彩粤剧文化周”活动成功举办，评选产生出富有文化底蕴的“佛山新八景”。教育改革和发展步伐加快。进一步理顺市、区两级教育管理体制，调整中小学布局结构，改造薄弱学校，优质普通高中打破地域界限，面向全市招生，高等教育进一步发展。科技、人才创新迈出新步伐。全市现有各类企业工程技术研发中心123家，其中省级31家；专业镇建设步伐加快，全市已建立15个专业镇；产学研合作广泛开展，建立企业博士后工作站11个。知识产权保护得到加强，专利申请量和授权量分别排名全省前列，推进全国专利试点工作，编订了《知识产权教育读本》，开展知识产权国民教育。信息化建设取得新成效，建设了一批信息化重点工程，全市信息化综合指数达73%。高新技术产业加快发展，全市现有高新技术企业203家，高新技术产品572个，高新技术产品产值730亿元，增长20.5%。卫生、体育工作取得新成绩。公共卫生体系建设步伐加快，卫生监督、疾病控制、妇幼保健、农村卫生和社区卫生服务取得新成效。全民健身运动广泛开展。按照“新省运、新佛山、新水平、新形象”和“精彩省运、活力佛山”的要求，第十二届省运会筹备工作紧锣密鼓展开。

——精神文明建设和社会各项事业再上新台阶。掀起学习贯彻十六大、十六届三中全会精神和“三个代表”重要思想的新高潮。以贯彻《公民道德建设实施纲要》为主线，以为人民服务为核心，以诚实守信为重点，加强公民道德建设。加强国防教育，强化了领导干部和市民的国防观念。民兵预备役、优抚安置和双拥工作得到巩固和发展，荣获全国双拥模范城“五连冠”。环境保护和计划生育工作在巩固中加强。以珠江综合整治为重点，制定《佛山市珠江水环境整治方案》，按照整治珠江“一年初见成效”的目标，生态环境保护和环保基础设施建设全面展开，工业污染源深化治理初见成效。加大国土资源管理力度，为经济建设提供用地保障。继续保持稳定低生育水平，人口自然增长率为3.72‰，控制在省下达的指标内。人民生活进一步改善。全面实施和落实“十项民心工程”。全市农民人均纯收入6603元，增长5%；城市居民人均可支配收入14827.2元，增长9.2%；年末城乡居民储蓄存款余额1783.99亿元，比年初增长16.2%。就业和再就业形势稳定。城镇登记失业率控制在2.3%以内，安排再就业资金5579万元，下岗失业人员安置率为76.6%。社会保障工作有新提高。企业退休人员社会化管理达到87%。“两个确保”继续巩固，全市参加养老保险119.5万人、失业保险89.5万人、医疗保险92.1万人、工伤保险120.6万人，比上年都有不同程度的增加。城乡最低生活保障基本实现“应保尽保”。

——民主法制建设和政府自身建设进一步加强。坚持向人大及其常委会报告工作，向政协通报情况，加强与各民主党派及工商联和工青妇等人民团体的联系，广泛征求对政府工作意见，促进政府决策的科学化和民主化。认真办理人大代表议案、建议和政协提案283件。行政执法监督不断加强，行政复议工作更加规范。社会稳定局面巩固。开展严打整治斗争，遏制各类刑事犯罪活动。严厉打击“法轮功”等邪教组织，推进“禁毒03工程”建设。完善了以110为龙头的社会援助机制，形成各级政府各部门共同参与的紧急援助体系。整顿和规范市场经济秩序，社会信用体系建设步伐加快。安全生产形势稳定，全国非公有制中小企业安全生产监督管理试点工作有序进行。政府职能进一步转变。简政放权取得突破性进展。市本级分三批向市辖各区下放了299项行政管理事项，将116项行政管理事项随职能划交（或委托）禅城区行使。开展了相对集中处罚权的试点工作。积极推进审批制度改革。市和各区都设立了行政服务中心或投资服务中心，实行“并联式”审批，提供“一条龙”服务，提高了行政效率。政府自身建设加强，开展以“三创新一优化”为主题的机关作风建设年活动，把工作重点放在更新治政理念、提高治政水平和加强政府服务上。推进电子政务建设，实行政务公开。廉政建设和反腐败斗争深入开展，廉政建设责任制进一步落实，在重大建设工程项目上，制定和完善预防职务犯罪的工作机制。

与此同时，残疾人事业、粮食储备、外事侨务、民族宗教、人防、档案、气象、保密、物价、统计、审计、广播电视、老龄及对口扶贫等各项工作都有新发展。

各位代表！过去一年成绩的取得，是党中央、国务院和省委、省政府及市委正确领导的结果，是市人大及其常委会和市政协监督支持的结果，是全市人民与广大建设者共同建设美好新佛山的结果，也是各民主党派、人民团体、驻禅部队、驻佛山直管单位、港澳台同胞、海外侨胞和外国友人大力支持的结果。特别是各区、镇（街）和工业园区都能把原有的基础优势以及区划调整后赋予的新“合力”和“活力”充分发挥出来，形成了“两‘虎’争先、五‘虎’生威”的经济发展新局面，使全市经济和社会事业呈现出加快发展、率先发展、协调发展的好势头，在此，我代表市政府对大家付出的努力和作出的贡献表示崇高的敬意和衷心的感谢！

在肯定成绩的同时也清醒地看到，我市经济和社会发展仍然存在不少困难和问题，主要表现在：产业水平不高，综合竞争力不强，产业发展后劲不足；投资环境尤其是软环境和可持续发展环境还不尽人意；制约经济社会发展的体制和机制性障碍仍然存在；在加快园区发展和城镇化进程中，“三农”问题仍有不少矛盾需要解决；文化发展相对滞后于经济社会发展，领导者和市民的文化素质有待提高，人文环境有待进一步改善；政府治政理念和职能转变还有不适应的地方等等。对这些问题，我们一定要增强忧患意识和紧迫感、使命感，采取有力措施，认真加以解决。

2004年的工作安排

2004年是深化改革、加快发展、狠抓落实的关键一年，是建设产业强市、文化名城和现代化大城市取得新成效的重要一年，也是各项建设全面展开之年。目前,佛山进入了一个新的发展机遇期，世界经济趋好,国内发展加快,我市各区、镇（街）和工业园区的积极性已充分调动起来；各项规划的确定，为下一步各项工作的全面展开奠定了基础；各项建设工程尤其是十大建设工程项目做了大量的前期准备工作，部分项目已经开始启动。这些都为我市做好今年工作创造了有利条件。但也要看到影响世界经济复苏的因素仍然存在；国内经济宏观政策在调整，经济发展的“瓶颈”仍然存在，加之周边兄弟市迅猛发展的强劲势头，进一步增大竞争压力。我们必须保持清醒的头脑，增强忧患意识，树立科学发展观，夯实发展基础，增强发展后劲，抓住机遇，乘势而上，努力实现更快更好发展。

2004年政府工作的指导思想是：以邓小平理论和“三个代表”重要思想为指导，全面贯彻党的十六大、十六届三中全会和胡锦涛总书记视察广东重要讲话精神，以“建设宽裕小康社会和率先基本实现社会主义现代化”为总目标总任务统揽全局，努力建设产业强市、文化名城和现代化大城市。按照市委九届三次全会确定的三项任务，坚持科学发展观，全力抓好各项工作落实。深化改革，扩大开放，优化结构，夯实基础，增强后劲，提升综合经济实力和国际竞争力。以人为本，加速城镇化进程，推进政治文明和精神文明建设，发展社会主义先进文化，提高人民生活水平，力促经济与社会协调发展、人与自然和谐发展，在我省实现向经济强省、文化大省的跨越中作出更大贡献。

2004年全市经济社会发展的主要预期目标是：生产总值增长12%；农业总产值增长3%；工业总产值增长18%；第三产业增加值增长11%；固定资产投资增长15%；社会消费品零售总额增长11%；出口总值增长8%；实际利用外商投资增长16%；地方财政一般预算收入增长10%；居民消费价格总水平比上年略有上升；城镇登记失业率控制在3.5%以内；人口自然增长率控制在6‰以内。

为实现上述目标，要认真做好以下工作：

一、以打造产业强市为目标，促进产业优化集聚提升

全力抓好工业园区建设。要按照“优化体制、加大整合、提升规模、整体竞争”的思路和“园镇互动”的体制，强化园区作为产业发展主战场的作用，以体制优势增强园区吸聚资源的竞争力。十大园区是重点，要在全力促进和扶持十大工业园区发展的同时，认真整合其它园区。按照产业特色化、园区品牌化的要求，完善园区规划和基础设施建设，推动园区整合和策划包装。利用我市国家、省高新区和若干产业基地的优势，以大企业、专业镇、名牌带动，引进产业关联度高、带动性强的龙头型骨干项目，加速产业链的形成，促进产业集群发展和优化提升。

做大做强支柱产业和龙头骨干企业。巩固提升家用电器、纺织服装、陶瓷及其他建材、金属材料及制品四大支柱产业；加快发展装备制造业，建设装备制造业基地；壮大发展食品饮料、塑料制品、精细化工及医药、家居用品制造四大潜力产业。打破地区、行业、所有制界限，引导骨干龙头企业优化资源配置，做大做强。鼓励、支持符合条件的企业到境内外上市，促进骨干企业与跨国公司尤其是世界500强合作，实现规模扩张。按照分类指导、层次推进的原则，制定完善的鼓励名牌发展的政策措施，扶持一批带动能力强、拥有自主知识产权的重点行业、重点企业和重点产品。

加快高新技术产业发展和传统产业改造。围绕产业强市的目标，加快国家和省的高新技术开发区和产业基地建设，大力发展电子信息、新材料、重大装备制造、数码光学、生物技术等高新技术产业，形成产业链，扩大产业规模，增强竞争优势。加快运用信息技术等高新技术和先进适用技术改造传统产业，通过技术改造和创新，形成新的竞争优势。

创新专业镇发展模式，促进中小企业发展。推进专业镇建设，重点抓好行业技术服务中心和生产力促进中心的建设，构建社会化科技服务网络，优化产业、产品和技术结构，打造专业镇品牌，形成专业化布局、专业化经营、市场化联动和社会化协作的新的专业镇发展格局。着力培育产业集群，搞好为大企业的协作配套，促进中小企业健康协调发展。要为专业镇和中小企业加快发展创新服务平台，建立和完善科技创新支撑体系。

推动以现代物流业为重点的第三产业发展。做好现代物流业发展规划，在重点园区、重要的产业聚集地和主要的交通、港口枢纽，高起点、高标准建设现代物流基地。推进连锁经营、电子商务、中高级批发市场等现代营销和流通方式，发展现代流通业。加快发展新兴服务业、旅游业、中介服务业和特色会展业。

加强区域协作。对外主动承接粤港等中心大城市特别是广州的产业、交通、金融、科技、人才等方面的辐射，主动承接广州汽车、石化和物流等产业延伸，提升发展水平和核心竞争力。对内根据产业发展规划和城市功能定位，制定行之有效的措施，推动东南部部分产业向西北部转移。

二、抓好招商引资，提升民营经济，促进内外源经济协调发展

创新招商引资机制，全力促进招商引资的规模和质量提升。充分发挥园区招商引资的载体作用，推动投资项目向园区集中。坚持以项目为中心，重点吸引世界500强企业、跨国公司和国内大企业在佛山聚集。继续抓好“9·26签约项目”的跟踪和落实，切实抓好项目的履约率、动工率，确保项目落实到位。建立政府招商机构和以中介组织为平台的民间招商机构，采取政府、企业、中介招商、以商引商等方式，全力抓好招商活动。通过与境外有影响的招商引资机构建立合作关系，通过双向交流达到引资目的。积极鼓励“三资”企业增资扩股，通过现有外商投资企业吸引、推荐上下游项目到各区、镇落户，形成完整的产业链条和产业聚集。同时，要运用行政力量加快推动重商气氛在全市形成，通过重商、亲商、稳商、安商、助商，进一步提高招商的吸引力和凝聚力。通过不断的努力，使外来投资在规模上有新发展，在质量和水平上有新提升。

大力发展民营经济，提高民营经济发展的质量和水平。落实好各项加快民营经济发展的政策措施，引导民营企业加快体制、科技和管理创新，走产权多元化和社会化道路。引导民营企业在参与国企改革、扩大产业投资和公用事业改革中做大做强。通过多种途径和形式，包括进入资本市场等促进民营企业做大做强；积极引导民营企业实行多元化股权结构等多种办法，促进建立现代企业制度。进一步放宽民营企业的市场准入和投资领域，让民营资本更广泛地进入三大产业投资领域。积极扶持和推动科技型、外向型、吸纳下岗人员再就业型、农产品加工型民营企业发展。采取有效措施解决民营经济发展中遇到的难题，通过建立和完善技术创新、市场拓展、筹资融资、人力资源、信息咨询服务等社会化服务体系，用更高效的服务、更低廉的投资经营成本和更好的软、硬环境，吸引更多的民营资本在我市聚集，促进民营企业更好更快健康发展。

落实内地与香港、澳门建立更紧密经贸关系安排，推动佛港澳经贸合作与发展。把握CEPA实施的发展机遇，加强与港澳货物贸易、服务业和投资便利化的更紧密合作，引导产业融入“粤港澳经济圈”。进一步优化营商环境，主动吸引港澳地区金融业、物流业、会展业、中介服务业、旅游业、商业零售业及房地产业等企业到我市经营发展。促进制造业与香港金融、物流、资金、技术的有机结合，提升我市制造业的整体素质。

加大对出口企业政策扶持力度，努力扩大出口。适应国家出口退税政策调整，制定配套政策和措施，推动出口稳步增长。调整优化出口产品结构，加大对本地出口产品的扶持，鼓励企业积极扩大本地产品出口，提高产品出口效益。建立与重点出口企业联系制度，鼓励重点出口企业增资扩产，扩大出口规模。鼓励有条件的企业“走出去”，开拓境外加工贸易，带动我市生产设备、原材料和零部件的出口。采取“请进来”和“走出去”的方式，举办和参加行业产品国际博览会，扩大产品出口渠道。民营企业办理申报进出口资格手续即到即办，支持民营企业开拓国际市场。

开拓国内市场，扩大消费需求。采取多种形式，引导和鼓励各类企业到内地省市开拓市场，加强与内地兄弟省市的经贸合作。积极培育和发展消费热点，大力发展假日经济、会展经济和旅游经济，鼓励扩大信息通讯、住房、汽车、社区服务、教育、文化、体育健身和卫生保健等领域消费。

三、认真解决农业农村农民问题，促进农业和农村经济发展

坚持用“三化”解决“三农”问题。贯彻落实好市委、市政府《关于加快农村工业化城镇化和农业产业化建设的决定》精神，创新体制和机制，加快农民变市民、农村经济社会形态变城市经济社会形态的转变，以工业化、城镇化带动农村劳动力转移，以农业产业化带动农业经济效益增长，提高农民收入。进一步完善提高以土地为中心的农村股份合作制，在保证农民的合法权益不受侵犯的条件下，促进农村生产要素的合理流动和优化组合。推进农村税费改革，强化农民负担监督管理，切实保护农民利益。积极探索和创新农村扶贫、“土地换社保”、农村集体经济和集体资产管理运行的新途径和新办法，建立城乡一体化就业服务网络，用城市救济模式解决贫困家庭、困难群众问题，让农民实现从依赖土地生存转到依赖就业和社会保障的跨越。

努力发展都市农业。以优质高效的都市农业为发展方向，调整优化农业结构。培育和发展农业龙头企业，推进农民专业合作经济组织建设，提高农民组织化程度。建设农业现代化示范园区，用现代高新技术改造农业，形成名优农产品优势区域布局。推行农业标准化生产，建立和健全农产品质量检验检测体系，推进农产品产地、产品认证和市场准入工作。进一步搞活农产品流通市场，发展农产品精深加工，提高农业效益。

加快农村城镇化进程。依照总体规划，抓紧编制农村城镇化规划，努力形成政府引导、科学规划、市场运作、产业支撑的城镇化机制。按省要求，抓好“并镇”、“并村”和“镇改街”、“村改居”工作，推进城镇簇群整合。以产业发展为依托，增强小城镇特别是中心镇吸纳农村人口、带动农村发展的能力。做好村镇建设试点，加大农民公寓建设和“城中村”改造力度。同时，改善农村基础设施，加大农田基本建设力度，高标准整治农田和鱼塘，提高土地利用率和土地产出率。

四、加大固定资产投资力度，全面展开现代化大城市建设

以“一环”干线为重点，全面推进主干公路和城际轨道交通建设。抓紧“一环”干线的施工设计、试验段工程、征地拆迁等各项工作，确保“一环”快速干线等22个项目今年全面动工；争取用三年左右时间完成“一环”等一批交通干线项目建设，初步形成组团式现代化大城市的主交通骨架。

全面启动中心组团新城区建设。在完成新城区基础设施建设的各项前期工作的基础上，加快体育场馆、新闻中心、东平大桥等项目的动工，确保中心组团新城区在2006年初现规模。

全力抓好电网和电源建设。要高度重视，落实责任，建立电网建设绿色通道，统一电网建设中征地、线行租用标准，妥善协调和积极解决电网建设中的问题，保障电网建设和改造工程的如期推进。同时，要加快电源项目建设，解决遏制经济发展“瓶颈”。

抓好现有道路交通环境的改善和建成区面貌的改造。制定好全市各区中心城区现有交通道路的整治规划，出台切实可行的整治措施和办法，疏导城际交通，改善交通秩序。整合交通资源，改革经营管理体制，推动公共交通的加快发展。加快各区中心城区面貌的治理和改造，改造中心城区出入口，使佛山以一个现代化大城市的崭新风貌迎接2006年省运会的召开。

推进城乡水利防灾减灾工程建设。以“水利三大会战”为契机，抓好城市防洪、江堤达标和小型水库安全加固建设。坚持城市防洪建设与道路交通、河道综合整治、道路绿化、休闲旅游和城市开发相结合，为市民创造安全舒适的生态水环境。

提高城市规划水平。按省的部署，认真搞好城镇化试点工作。在城市发展概念规划指导下，按照组团式现代化大城市发展的要求，完成市域城镇体系规划，两个百万人口、五个三十到五十万人口的新城区规划，以及市域历史文化名城保护规划和园林绿地系统规划，完善、修订和推进中心镇规划，确保市、区两级规划的衔接配套和功能布局合理。制订严格的规划管理制度，确保各项建设按规划要求组织实施。

五、深化改革，构筑经济社会发展体制新平台

深化产权制度改革。通过多种形式加快公有企业的改造、完善职工社会保障、妥善处理经济补偿、加快劣势公有企业退出市场等一系列改革，为增强企业活力和竞争力创造条件。建立有效促进国有资产整合、运营和增值的新型国有资产监管体制，抓好市级公有资产管理机构的设立。完善企业经营业绩考核体系和经营者的激励监督机制，促进政府公共管理职能和国有资产出资人职能分离。加强公有资本整合和重组，引进投资者改造基础性、垄断性领域的产权结构，优化企业股权结构，通过努力，将公有企业改造成为投资主体多元化、运营规范化的新型现代企业。

推进投融资体制改革。按照“谁投资、谁得益、谁承担风险”的原则，多元化、市场化筹措资金，确保十大建设工程和重点项目建设资金。积极探索政府资源市场化和城市可经营项目投资主体多元化运作办法，建立市场引导投资、企业自主决策、融资方式多样、中介服务规范、宏观调控有效的新型的投融资管理体制。按照“投资多元化，运营市场化，服务社会化，发展产业化”的思路，逐步推进公共交通、污水处理、垃圾处理等市政公用事业和城市经营项目的社会化、市场化进程。创新固定资产投资机制，引入市场竞争机制，凡是可以让投资者参与的重大基础建设项目，都将向全市、全省，向境内外开放，吸引民间、外地和境外资本参与投资建设经营，实现固定资产投资多元化和社会化。

探索城乡户籍管理制度改革。今年要统一全市各类户口为居民户口，实行本市居民以实际居住地登记户口，统一户口准入条件，待条件成熟时实行五区户口互通的管理模式。在此基础上，逐步将依附在原有户籍管理制度上的计生、教育、就业、社会保障等一系列的管理制度进行改革与创新。

推进社会管理体制改革。围绕“同城生活、同城便利”的目标，搞好公用事业的开放与改革，实现同城范围内基础设施、文体教育医疗、城市应急机制和社会服务无缝对接。加快和规范产业行业协会、商会等中介组织的发展，发挥其在经济社会发展中的桥梁和纽带作用。进一步强化街道、社区、村委会组织的建设，深化社区居民委员会改革，探索建立城市基层组织管理机制和运行机制，逐步探索和稳步推进“村改居”工作。此外，积极推动机关后勤事务社会化，探索公务用车等职务消费的改革。

加快人事制度改革。认真做好新一轮政府机构改革，推进事业单位改革，铺开事业单位工作人员聘用制工作。实施“人才强市”战略，牢固树立人才资源是第一资源的观念，创新人才机制，优化人才环境，加大人才引进力度。实施“优秀人才培养工程”，奖励有突出贡献的人才。充分发挥人才的积极性、主动性和创造性，为全面建设宽裕小康社会和现代化大城市提供坚强的人才保证和智力支持。

六、推动文化名城建设，促进经济社会协调发展

加强文化名城建设，提高公民思想道德素质。以提高市民素质和城市文明程度为目标，以文化名城建设和承办省运会为契机，进一步加大精神文明建设力度。弘扬佛山人文精神，塑造佛山新形象。深化以诚信为重点的公民道德建设，以创建“省级文明城市”为动力，扎实开展文明城区、文明村镇、文明行业和文明单位等群众性精神文明创建活动。抓好国防教育，提高国防动员和民兵预备役工作水平，做好双拥共建和优抚安置工作，巩固双拥模范城成果，推动双拥工作向基层延伸，向社会各界拓展。深化文化体制改革，繁荣文化事业，发展文化产业。实施建设文化名城的战略，推进特色优势文化产业化进程。挖掘、整理、提升历史文化资源，打造具有佛山特色和浓郁文化底蕴的陶艺、粤剧、武术、民间艺术、中医药、饮食等文化品牌。搞好广东粤剧博物馆的建设和完善，抓紧建设市新闻中心等一批标志性大型文化基础设施。整合、提升现有文化设施，加强文化名镇、名村、名馆、名园、名景的保护和建设。广泛开展丰富多彩的文化活动，提升城市文化形象。

推进“教育强市”建设。加大教育投入，落实基础教育“以区为主”管理体制，加快中小学布局调整，加大等级学校建设力度，高质量、高水平普及高中阶段教育，大力发展优质教育，全面推进教育强区、强镇建设。以素质教育为重点，深化义务教育和高中阶段教育的新课程改革，加快教育信息化建设，全面提高教育质量。大力发展职业教育，确保普通教育与职业教育协调发展。深化办学体制改革，鼓励和规范社会力量办学。加快高等教育资源整合和佛山大学园区规划建设。完善现代国民教育体系和终身教育体系，创建学习型社区，促进学习型社会的形成。加强教育督导，推进依法治教。

促进科技创新。完善科技创新服务体系，增强企业科技创新能力，加快国家、省高新产业开发区和特色产业基地建设，推动园区和基地科技孵化器建设，制订和实施知识产权发展战略，加大知识产权保护力度。加强生产力促进中心和院士、博士后工作站（室）建设，广泛开展产学研合作。进一步推进信息化建设。加快建设高速率、大容量、高可靠性的基础传输网，推进基础数据库建设。继续推进制造业信息化工程建设。整合信息资源，拓展信息技术在经济社会各方面应用范围，提高信息资源使用效率，全面提升国民经济和社会信息化水平。

建立和健全公共卫生管理体系。引进民资、外资发展医疗产业。加强疾病预防控制、卫生执法监督和医疗救助体系建设，提高疾病预防控制和应对突发公共卫生事件能力。完善医疗机构分类管理，深化医疗机构运行机制改革和医院产权制度改革。加快公共卫生设施建设，加强妇幼卫生工作，完善社区卫生网络，加快建立新型农村合作医疗制度。深入开展全民爱国卫生运动，改善城乡卫生环境。高度重视和预防冬春两季传染病的发生，按照中央和省的部署，突出抓好防治非典、禽流感和其它传染病的工作。

促进体育等各项事业发展。全面推进第十二届省运会各项筹备工作，营造迎接省运会的良好软、硬环境。整合体育人才资源，提升竞技体育水平。大力开展群众性体育运动，构建多元化全民健身服务体系。深化体育事业改革，发展壮大体育产业。继续做好外事侨务、台务、档案、民族宗教、妇女儿童、老龄等各项工作。

七、加强环境资源保护和计划生育工作，促进可持续发展

加强环境保护。强化环保法规和政府环保责任制的落实，抓好《佛山市可持续发展的生态环境规划纲要》的落实，全面推动创建国家环保模范城市的各项工作，按照省确定的珠江整治目标任务，抓紧推进整治计划和工程建设项目，加大佛山水道和内河涌的整治力度，改善流域水质，提高水环境质量，为实现“三年不黑不臭”的目标打下基础。加快环保基础设施建设，抓好生活垃圾、生活和工业污水处理，加强工业园区环境规划和管理，从源头上控制新污染源的产生。推广使用清洁能源和污染防治新技术，加强城市大气污染综合整治，着重治理机动车尾气及重点行业二氧化硫和粉尘污染的防治，严格控制噪声污染。

抓好生态环境保护。加大水源保护区、自然保护区、生态公益林和风景名胜区的保护力度，做好创建生态示范区、生态示范村、镇、园工作。加快城市绿化系统、城市组团之间绿地隔离带和生态屏障建设，不断建设绿色社区。加大农业环境污染治理力度，依法保护和合理开发自然资源。严格实施土地用途管制，坚决遏制乱占耕地、林地。加快建立和完善土地储备制度，加大力度处置闲置土地，促进土地的合理、高效和可持续利用。

加强人口与计划生育工作。继续稳定低生育水平，提高出生人口素质。加强计划生育基层组织建设，确保工作重心下移到基层。适应加快农村城镇化进程、户籍制度和婚姻登记制度改革等要求，探索人口与计划生育工作新机制。

八、落实好十项民心工程，改善人民生活质量

切实关心群众生活。实施好十项民心工程，努力为群众多办实事、好事，加强对各级的考核，确保各项民心工程落实到位。改善人民生活质量，切实解决好困难群众吃、住、行、看病、上学、打官司等问题。

完善社会保障制度。完善现行城镇基本养老、医疗、工伤、失业等保险制度及住房分配货币化、住房公积金制度，扩大覆盖面，并按省统一部署，实现养老保险市级统筹。抓好个体私营等非公有制企业和灵活就业人员的参保工作，做好下岗职工基本生活保障与失业保险并轨的后续工作。在农村，加快全面建立合作医疗或基本医疗保险制度，有条件的地方要积极探索建立基本养老保险制度。

扩大和促进就业。积极落实就业和再就业的各项政策，确保就业和再就业工作目标的实现，努力扩大就业，促进再就业。结合改革城乡二元结构，抓好城乡劳动力就业服务一体化平台的建立，促进农村劳动力向城镇二、三产业转移。严格贯彻执行《劳动法》，依法维护外来员工合法权益。

九、依法征税理财，努力增收节支

加强税收征管，积极组织收入。抓好经济发展，努力培育地方税源。规范非税收入管理，完善国有资产收益收缴管理制度。严格依法治税，加大征收稽查力度，严厉打击偷税逃税等违法犯罪活动，确保税收及时足额入库。同时，认真落实国家减免税政策，扶持重点产业和行业的发展。

规范财政管理，优化支出结构。增收节支，严格预算管理，集中财力保稳定、保安全、保运转、保改革和保重点，加大对农业投入，支持产业强市、文化名城和现代化大城市建设以及“十项民心工程”建设的需要，努力使财力向社会管理和公共服务倾斜。对日常经费开支要严格控制，今年市本级财政对预算内、外资金安排的一般性支出除法律法规规定增长外，一律按零增长安排。

强化财政监督，促进依法理财。严格部门预算，完善政府采购制度，进一步落实“收支两条线”管理规定，加强审计监督，加大财务总监工作力度，确保财政资金完整、安全、高效运行。同时，要深化各项财政改革，积极构建公共财政体系，加快推进国库集中支付改革试点，积极开展财政支出绩效评价试点工作。要认真用好财政资金，发挥财政资金在公共管理和建设中“四两拨千斤”作用。

十、坚持依法行政，推进民主法制建设

加强民主法制建设。坚持向人大及其常委会报告工作，向人民政协通报情况制度，自觉接受监督。认真办好人大代表议案、建议和政协提案。建立和完善重大事项社会公示和听证制度、专家咨询制度、决策责任制度，多方听取各民主党派、工商联、人民团体和社会各界的意见和要求，提高决策的透明度和公众参与度，推进决策的民主化、科学化、制度化。健全基层自治组织，进一步推进村民自治和社区居民自治。加强行政监督，规范行政行为。

维护社会稳定。严密防范和严厉打击敌对势力、“法轮功”邪教组织的破坏活动和暴力恐怖活动。贯彻“打防结合、预防为主”的方针，坚决打击各种刑事犯罪和经济犯罪活动，深入推进“禁毒03工程”，扫除社会丑恶现象。加强社会治安综合治理，推进社会治安防控体系建设。积极开展人民内部矛盾纠纷排查调处工作，做好人民来信来访工作，努力化解各种社会矛盾和纠纷，依法、妥善处理群体性事件，保持社会和谐稳定。加强安全生产监督管理，整治安全隐患，建立安全生产监管长效机制，做好全国非公有制中小企业安全生产监督管理试点和推广工作。

加强廉政勤政建设。开展“两个务必”教育，落实“五个力戒”和“干净干事”要求。坚持标本兼治、综合治理、注重预防的方针，推进从源头上预防和治理腐败的工作，建立健全与社会主义市场经济体制相适应的教育制度，监管并重的惩治和预防腐败体系。加快构建科学、严密的权力运行机制，进一步规范事权、财权和人事权。坚持不懈推进反腐败斗争，提高自律意识和遵纪守法的自觉性，加大对违法违纪案件的查处力度。加强行政监察特别是行政效能工作，提高行政运行效率。加强对财政资金、国有资产和重大工程项目的审计监督，狠刹部门和行业不正之风。

切实更新政府治政理念。进一步转变政府管理方式，提高治政水平。加大“放权于基层”、“还权于社会”的力度。抓好简政放权工作的检查落实，确保下放的管理项目完全到位。进一步明确划分市、区、镇三级政府的事权和管理职责。同时，要加快将政府部分职能交由产业性行业协会（商会）承担。市本级政府，重点把握好关系全市发展大局的重大改革、重大发展、重大基础设施建设的规划、策划、监管和服务，把事务性的管理权下放基层。推进政府管理方式创新，推行政务公开，推进电子政务建设，利用现代信息技术改进政府的组织和管理，及时把政府决策、服务程序、办事方法向社会公布，为人民群众提供公开、透明、高效的公共服务。

进一步提高依法行政水平。认真做好《行政许可法》的学习、宣传、培训，提高公务员素质，采取有效措施，促进《行政许可法》的落实。制订《佛山市国家公务员服务行为规范暂行规定》，规范公务员行政行为。加强政府法制建设，实行行政执法责任制和过错追究制，设立行政投诉中心，完善行政监督制度，加快建立权责明确、行为规范、监督有效、保障有力的执法体制。创新行政管理，转变政府职能和作风，形成层次少、服务好、效率高的政务环境。加大城市管理行政执法力度，推进相对集中处罚权综合执法工作，提高城市管理水平。

各位代表！我们肩负着建设宽裕小康社会和现代化大城市的神圣使命，任务繁重而艰巨。我们一定要在邓小平理论和“三个代表”重要思想指导下，全面贯彻十六大和十六届三中全会精神，树立强烈的事业心、责任感和使命感，与时俱进，开拓进取，团结奋斗，努力谱写佛山改革开放和现代化建设的新篇章！
